# Comparison of explanatory and pragmatic design choices in a cluster-randomized hypertension trial: effects on enrollment, participant characteristics, and adherence

**DOI:** 10.1186/s13063-022-06611-3

**Published:** 2022-08-17

**Authors:** Karen L. Margolis, A. Lauren Crain, Beverly B. Green, Patrick J. O’Connor, Leif I. Solberg, MarySue Beran, Anna R. Bergdall, Pamala A. Pawloski, Jeanette Y. Ziegenfuss, Meghan M. JaKa, Deepika Appana, Rashmi Sharma, Amy J. Kodet, Nicole K. Trower, Daniel J. Rehrauer, Zeke McKinney, Christine K. Norton, Patricia Haugen, Jeffrey P. Anderson, Benjamin F. Crabtree, Sarah K. Norman, JoAnn M. Sperl-Hillen

**Affiliations:** 1 HealthPartners Institute, Mailstop 21112R, PO Box 1524, Minneapolis, MN 55440-1524 USA; 2 Kaiser Permanente Washington Health Research Institute, 1730 Minor Av, Seattle, WA 98101 USA; 3 Rutgers Robert Wood Johnson Medical School, Department of Family Medicine and Community Health, New Brunswick, NJ 08901 USA

**Keywords:** Hypertension, Self-measured blood pressure, Telemonitoring, Pharmacist care, Pragmatic trials

## Abstract

**Background:**

Explanatory trials are designed to assess intervention efficacy under ideal conditions, while pragmatic trials are designed to assess whether research-proven interventions are effective in “real-world” settings without substantial research support.

**Methods:**

We compared two trials (Hyperlink 1 and 3) that tested a pharmacist-led telehealth intervention in adults with uncontrolled hypertension. We applied PRagmatic Explanatory Continuum Indicator Summary-2 (PRECIS-2) scores to describe differences in the way these studies were designed and enrolled study-eligible participants, and the effect of these differences on participant characteristics and adherence to study interventions.

**Results:**

PRECIS-2 scores demonstrated that Hyperlink 1 was more explanatory and Hyperlink 3 more pragmatic. Recruitment for Hyperlink 1 was conducted by study staff, and 2.9% of potentially eligible patients enrolled. Enrollees were older, and more likely to be male and White than non-enrollees. Study staff scheduled the initial pharmacist visit and adherence to attending this visit was 98%. Conversely for Hyperlink 3, recruitment was conducted by clinic staff at routine encounters and 81% of eligible patients enrolled. Enrollees were younger, and less likely to be male and White than non-enrollees. Study staff did not assist with scheduling the initial pharmacist visit and adherence to attending this visit was only 27%. Compared to Hyperlink 1, patients in Hyperlink 3 were more likely to be female, and Asian or Black, had lower socioeconomic indicators, and were more likely to have comorbidities. Owing to a lower BP for eligibility in Hyperlink 1 (>140/90 mm Hg) than in Hyperlink 3 (>150/95 mm Hg), mean baseline BP was 148/85 mm Hg in Hyperlink 1 and 158/92 mm Hg in Hyperlink 3.

**Conclusion:**

The pragmatic design features of Hyperlink 3 substantially increased enrollment of study-eligible patients and of those traditionally under-represented in clinical trials (women, minorities, and patients with less education and lower income), and demonstrated that identification and enrollment of a high proportion of study-eligible subjects could be done by usual primary care clinic staff. However, the trade-off was much lower adherence to the telehealth intervention than in Hyperlink 1, which is likely to reflect uptake under real-word conditions and substantially dilute intervention effect on BP.

**Trial registration:**

The Hyperlink 1 study (NCT00781365) and the Hyperlink 3 study (NCT02996565) are registered at ClinicalTrials.gov.

## Introduction

Explanatory trials are intended to test interventions with the main goal of demonstrating their efficacy under ideal conditions, while pragmatic trials are intended to test whether research-proven efficacious interventions are effective in “real-world” settings without extra research support for recruitment, adherence, retention, and fidelity to interventions. Clinical trials should be designed to fit their specific purpose and can be scored across nine domains on a continuum from “very explanatory” to “very pragmatic” using the PRagmatic Explanatory Continuum Indicator Summary-2 (PRECIS-2) tool [1]. Key decisions facing trialists include selecting suitable options in each of these domains: setting, recruitment, eligibility, organization (expertise and resources needed to deliver interventions), flexibility of delivery, flexibility of adherence, follow-up, primary outcome, and primary analysis.

In this paper, we illustrate some of the major differences between explanatory and pragmatic clinical trial designs using two projects that addressed the same research question: to compare the effects on blood pressure (BP) of (1) a pharmacist-led telehealth intervention in adults with uncontrolled hypertension with (2) clinic-based primary care. Home BP monitoring combined with care management by a pharmacist or nurse has been shown to lower BP and improve control of hypertension [2–14]. Like most of the studies in this evidence base the Hyperlink 1 cluster-randomized trial was designed to show efficacy under relatively tightly controlled conditions, although it had broader eligibility criteria than trials that preceded it [15]. In Hyperlink 1, patients with uncontrolled hypertension who received home BP telemonitoring combined with pharmacist-led telephone care safely achieved 10/5 mm Hg greater BP reduction over 12 months compared with patients who continued to receive routine primary care [16]. The large intervention effect prompted the study team to undertake further research to see if the Hyperlink 1 results could be implemented in the same setting by usual primary care staff without direct involvement by research staff.

Thus, Hyperlink 3 was designed as a larger-scale comparison of the telehealth intervention program with clinic-based care and it intentionally included many more pragmatic design elements. This paper compares the explanatory and pragmatic design choices in Hyperlink 1 and Hyperlink 3 and illustrates how the pragmatic design of Hyperlink 3 affected enrollment, participant characteristics, and adherence to the study interventions in important ways. The comparison highlights some of the dilemmas in designing pragmatic trials and interpreting their results.

## Methods

The methods for the two trials have been described in detail [15, 17], with key points summarized in Table 1. Both were cluster-randomized trials in the same health system with primary care clinics as the unit of randomization to reduce risk of contamination if clinic physicians cared for patients in both the intervention and control group. The focus here is on describing the differences in explanatory vs. pragmatic design choices, with particular reference to the nine elements in the widely used PRECIS-2 tool: setting, recruitment, eligibility, organization (expertise and resources needed to deliver the intervention), flexibility of the intervention, flexibility of adherence to the intervention, follow-up, primary outcome, and primary analysis [1]. Hyperlink 1 and 3 were each initially scored by the principal investigator along the continuum from very explanatory (1 point), rather explanatory (2 points), equally pragmatic/explanatory (3 points), rather pragmatic (4 points), and very pragmatic (5 points). The initial scores were reviewed by 2 co-investigators and discrepancies were resolved by consensus.

**Table 1:** Comparison of pragmatic design elements in Hyperlink 1 and Hyperlink 3

Design element	Hyperlink 1	Hyperlink 3
Patients enrolled	450	3071
Setting	16 primary care clinics with • MTM pharmacists	21 primary care clinics with • MTM pharmacists • Automated BP monitors
Recruitment period	Mar 2, 2009–Apr 29, 2011	Nov. 15, 2017–Apr 16, 2019
Recruitment method	Mailings, telephone screening, and final determination of BP eligibility, informed consent, and enrollment at research clinic	Automated EHR algorithm for screening at primary care encounters, prompted staff and PCPs to complete follow-up orders for enrollment
Eligibility	Age 21 or older 2 most recent ERH BPs >140/90 Ave of 3 research clinic BPs • >140/90 or • >130/80 if DM or CKD Major exclusions: • Pregnancy • Recent MI or stroke • Stage 4/5 CKD • Stage 3/4 heart failure	Age 18–85 2 most recent EHR BPs >150/95 Hypertension diagnosis PCP visit in last 12 months Major exclusions: • Pregnancy • Stage 5 CKD • Hospice • Nursing home resident
Comparator name	Usual care	Best practice clinic-based care
Intervention name	Telemonitoring, pharmacist care management	Telehealth care
Organization (expertise and resources needed to deliver interventions)	For usual care, no additional expertise or resources; for telemonitoring, 8 h of pharmacist training, telemonitors paid for by study funds	For clinic-based care, no additional expertise or resources; for telehealth, 3.5 h of pharmacist training, telemonitors paid for by health system
Flexibility of delivery	For usual care, very flexible; for telemonitoring, pharmacists followed protocol in addition to collaborative practice agreement	For clinic-based care, very flexible except initial follow-up recommended with medical assistant; for telehealth care, pharmacists followed protocol allowing more individualized care
Flexibility of adherence	For usual care, routine attention to adherence; for telemonitoring, patients assisted with appointments and pharmacist encouraged adherence to intervention.	For clinic-based care, routine assistance with initial appointment; for telehealth care, routine assistance with initial appointment, but pharmacist encouraged adherence to intervention.
Follow-up and data collection	Research clinic at 0, 6, 12, and 18 months for all participants to measure BP and administer surveys	No research clinic visits BP data extracted from routine visits in EHR, surveys for data not in EHR
Primary outcome	BP control at 6 and 12 months	Change in SBP from baseline to 12 months
Primary analysis	Intention-to-treat	Intention-to-treat

*Abbreviations*: *MTM* Medication therapy management, *BP* blood pressure, *EHR* electronic health record, *DM* diabetes mellitus, *CKD* chronic kidney disease, *MI* myocardial infarction, *PCP* primary care professional, *SBP* systolic blood pressure

### Setting

Both Hyperlink trials were conducted at HealthPartners, a nonprofit integrated health system in Minnesota and western Wisconsin serving 1.8 million health plan members and 1.2 million patients with a wide variety of insurance coverage. HealthPartners expanded by acquiring several health systems between the two trials, thus expanding the number of potentially eligible clinics. It currently includes a multispecialty group practice of more than 1800 physicians, 25 medication therapy management (MTM) pharmacists, eight hospitals, and 55 primary care clinics. Clinics were eligible to participate in both trials if they had an MTM pharmacist onsite at least one half-day per week. Hyperlink 3 had the additional eligibility requirement that clinics use standardized methods to measure BP with validated oscillometric BP monitors at the time of study startup. In both trials, all eligible clinics agreed to participate (16 clinics in Hyperlink 1 and 21 clinics in Hyperlink 3). Hyperlink 1 started in 2009 and Hyperlink 3 in 2017.

### Recruitment of patients

In Hyperlink 1, recruitment was carried out by research staff, starting with identifying 15,549 potentially eligible patients based on BP recorded in the electronic health record (EHR) during care, followed by postal mailings and telephone and research clinic screening for eligibility and recruitment of interested respondents by research staff. Patients were enrolled after meeting the eligibility criteria and giving informed consent.

In Hyperlink 3, the goal was to enroll patients during a primary care visit without the need for research staff and thus to enroll patients who were representative of the entire eligible target population. Eligibility for Hyperlink 3 was evaluated using automated real-time algorithms that were triggered upon BP entry into the EHR during primary care office encounters in study clinics. For eligible patients, a best practice alert automatically prompted the medical assistant to set up a referral order for hypertension follow-up for the primary care professional (PCP) to review and sign. The referral order defaulted to in-person follow-up in 1–2 weeks depending on the clinic’s randomization status (medical assistant BP check for clinic-based care and MTM pharmacist for telehealth clinics, with PCP or specialist as additional non-default options). The medical assistant and PCP were encouraged but not required to follow through on the referral prompts. PCPs were able to change the type or timing of follow-up from the defaulted choice on the referral order if they felt that a different choice was best for an individual patient. Telehealth care with home BP telemonitoring was only available for patients in telehealth clinics and was offered to patients at a subsequent MTM pharmacist visit. Enrollment in Hyperlink 3 was defined by an eligible patient having a signed hypertension referral order regardless of whether the default follow-up was kept or changed. The enrollment date was the primary care encounter during which the referral was signed.

The HealthPartners Institutional Review Board (IRB) reviewed both study protocols. For Hyperlink 1, written informed consent was included in the protocol and obtained at the first research clinic visit. For Hyperlink 3, the IRB approved a partial waiver of informed consent for enrolling patients in the study because (1) the interventions did not pose additional safety risks compared to routine care for hypertension, and (2) the study could not have been practicably carried out had written informed consent been required. Enrolled patients were later contacted to complete surveys and the mailed cover letter and telephone scripts included the elements of informed consent, including that completing the survey implied their consent to use their data in the study.

### Eligibility criteria for patients

Eligibility for Hyperlink 1 started at age 21 with no upper age limit. Patients were considered potentially eligible and were sent recruitment letters if they had at least two primary care outpatient encounters in the previous 12 months with BP >140/90 mm Hg at the two most recent visits. Further screening by telephone and medical record review excluded patients who were pregnant or had recent vascular events, advanced kidney disease, or heart failure. The average of three BP standardized measurements in the research clinic had to be >140/90 mm Hg or >130/80 if the patient had diabetes or chronic kidney disease (CKD). There was no explicit exclusion for hospice or nursing home residence.

Most of the eligibility criteria for Hyperlink 3 were chosen to reflect the denominator population that is included for hypertension quality measures [18, 19]. Patients were potentially eligible if they (1) were age 18 to 85; (2) had two or more encounters with a hypertension diagnosis code within the last 24 months; and (3) had a visit with their assigned PCP in the last 12 months and were currently at a visit in the clinic where their assigned PCP practiced. Study criteria for uncontrolled BP were defined as SBP > 150 mm Hg or DBP > 95 mm Hg in the first BP and in a repeated BP within the current encounter as well as the last recorded BP at the most recent previous encounter. The higher BP criteria for Hyperlink 3 for uncontrolled BP were rooted in the pragmatic design and were based on the estimated sample size (at least 1000 per randomized group), the estimated number of eligible patients per clinic, and the capacity of MTM pharmacists in telehealth clinics to accommodate additional follow-up referrals. Study exclusions were also similar to hypertension quality measures: pregnancy, stage 5 CKD, hospice care, and permanent nursing home residence.

### Organization

#### Controls: usual care (Hyperlink 1) and best practice clinic-based care (Hyperlink 3)

In Hyperlink 1, there was no effort to describe systematically or influence usual care (UC) for hypertension. Routine primary care at that time included options for a PCP to order conventional home BP monitoring, no-cost BP check visits with a medical assistant, and referral to MTM pharmacists for in-person consultations without prolonged monitoring or telephone follow-up. There was monthly feedback to physicians regarding BP levels in their patients with diabetes, and promotion of then-current national and regional guidelines for hypertension management [20, 21].

In Hyperlink 3, in recognition of improvements in hypertension care that had been adopted since Hyperlink 1, the comparator was called “best practice clinic-based care.” The best practices were recommended by professional organizations (e.g., American Medical Association, American Medical Group Association) at the time the study was designed and were affirmed in subsequent national guidelines [22]. New infrastructure and policies in place at HealthPartners that promoted high-quality care in 2017 included the following components: (1) measurement of all BPs using validated oscillometric BP monitors (Omron HEM 907XL) [23] according to a standard nursing protocol (in the subset of clinics eligible for the study); (2) a hypertension registry to identify and track the patient population with hypertension and systematically recall those with uncontrolled BP for additional BP checks with a medical assistant or PCP; (3) monthly feedback on PCP and clinic performance on BP measurement and control for patients with diabetes, vascular disease, or hypertension; (4) endorsement of an evidence-based hypertension treatment guideline that promoted low-cost generic medication, single-pill combination therapy, and follow-up at 2–4-week intervals until BP is controlled; and (5) no-cost BP check visits with a medical assistant with a standing order protocol for registered nurses to adjust hypertension treatment for uncontrolled BP. The best practice clinic-based intervention reflected standard workflows to the extent they were followed by each clinic.

#### Interventions: telemonitoring with pharmacist care management (Hyperlink 1) and telehealth care (Hyperlink 3)

The Hyperlink 1 intervention is described in detail elsewhere [15]. Briefly, patients met with a pharmacist for an initial in-person assessment visit and then received a home BP telemonitoring kit delivered to their home. They were asked to transmit at least 6 BP measurements weekly. During the first 6 months of the intervention, telephone visits with a pharmacist were to occur every 2 weeks by telephone until BP control was sustained for 6 weeks, and then frequency was reduced to monthly. During intervention months 7 through 12, telephone visits with a pharmacist were to occur every 2 months. Pharmacists advised lifestyle changes, encouraged medication adherence, and adjusted antihypertensive drug therapy using a collaborative practice agreement. The protocol suggested medication intensification at each telephone visit if fewer than 75% of measurements met the home BP goal (<135/85 mm Hg, or <125/75 mm Hg for patients with diabetes or chronic kidney disease.) All patient encounters were documented in the EHR and sent electronically by the pharmacist to the PCP; informal in-person communication and consultation also occurred. The intervention lasted 12 months, and patients were then advised to then resume usual care with their PCP.

The telehealth care intervention in Hyperlink 3 was similar to the Hyperlink 1 intervention, but differed in the following respects [17]. (1) Routine primary care included all of the best practices that had been adopted to improve hypertension care since Hyperlink 1. (2) In one telehealth care clinic with very limited MTM pharmacist availability, two nurse practitioners took on the pharmacist role. (3) The home BP goal was <135/85 mm Hg for all patients. (4) The duration of the intervention was individualized rather than fixed in length. (5) All patient contacts were conducted by usual clinic personnel, not research staff. Patients were asked to continue telemonitoring and pharmacist telephone visits until BP was controlled, defined as three or more consecutive visits at least 2 weeks apart with ≥75% of home BP measurements below 135/85 mm Hg, with the expectation that the average duration would be about 4 months based on experience in Hyperlink 1. Patients could also be discharged from telehealth if they were persistently non-adherent with home monitoring or phone visits, became unreachable, or the pharmacist and patient agreed that telehealth care was unlikely to result in further BP lowering. (5) When telemonitoring was completed, patients were supplied with a non-transmitting version of the home BP monitor for continued use. (6) Pharmacists were encouraged to manage other risk factors including smoking, hyperlipidemia, and hyperglycemia in diabetic patients using evidence-based protocols in their collaborative practice agreements.

#### Expertise and resources needed to deliver interventions

As noted above in the section on recruitment, study resources went into identifying and enrolling eligible participants for both studies. Hyperlink 1 UC and Hyperlink 3 clinic-based care required no additional expertise or resources to deliver the interventions. For the Hyperlink 1 telemonitoring intervention, pharmacists received 8 h of study protocol training plus 1:1 observation of their first study patient visit, their labor was paid for by study funds, and the telemonitoring was also paid for by study funds. For the Hyperlink 3 telehealth care intervention, pharmacists received 3.5 h of study protocol training plus 1:1 observation of their first patient visit, and both their labor and the telemonitoring was paid for by the health system. In addition, study staff attended part of monthly MTM pharmacist staff meetings for ongoing communication about study issues and progress.

### Flexibility of delivery

Hyperlink 1 UC and Hyperlink 3 clinic-based care were very flexible and pragmatic, except that the Hyperlink 3 default initial follow-up recommendation was for a medical assistant BP check. For the Hyperlink 1 telemonitoring intervention, pharmacists were asked to follow a fixed visit schedule and protocol for medication intensification that was based on the existing collaborative practice agreement. For the Hyperlink 3 telehealth care intervention, the pharmacists were allowed more flexibility in the visit schedule, intervention duration, medication intensification approach, and management of conditions in addition to hypertension.

### Flexibility of Adherence to Intervention

Hyperlink 1 UC patients had routine attention to adherence and no additional assistance with scheduling follow-up. For the Hyperlink 1 Telemonitoring Intervention, research staff scheduled enrolled patients for the initial in-person pharmacist visit but phone follow-up visits were scheduled by the pharmacists. The pharmacists encouraged telemonitoring, medication, and phone visit adherence.

In Hyperlink 3, any assistance patients in both intervention groups received with scheduling the initial in-person follow-up- visit was from clinic staff according to workflows and capacities that varied by clinic and over time. Enrolled patients in clinics randomized to clinic-based care were given a signed referral order to follow up with a medical assistant for a BP check within 2 weeks, or another type of follow-up if the PCP had changed the default. Patients could schedule their follow-up visit in the clinic or by phone. Clinic assistants used a “referral work queue” to place up to two phone calls to reach non-scheduled patients and then sent a letter to non-responders. Any further follow-up and attention to adherence was at the discretion of the patient and care team, but patients with uncontrolled BP could also be contacted by staff from either the clinic or a centralized resource to schedule BP follow-up through the hypertension registry. Patients in clinics randomized to telehealth care had a signed referral order to follow up with a pharmacist within 2 weeks, or another type of follow-up if the PCP had changed the default. Follow-up scheduling in telehealth care clinics was similar to clinic-based care clinics except that an MTM program coordinator used the referral work queue to schedule the initial in-person pharmacist visit. For patients who attended the initial in-person pharmacist visit, pharmacists encouraged uptake and adherence to telemonitoring, as well as both medication and phone follow-up visit adherence.

### Follow-up for data collection

In Hyperlink 1, research clinic follow-up visits were scheduled at 6, 12, 18, and 54 months following enrollment to measure BP and administer surveys for patient-reported outcomes. In Hyperlink 3, there were no research visits. BP measures were extracted from routine visits in the EHR and patient-reported outcomes were collected in surveys conducted by telephone, mail, or online at baseline, 6, 12, and 24 months.

### Primary outcome relevance to patients

In Hyperlink 1, the primary outcome was BP control at 6 and 12 months as measured in research clinic visits. In Hyperlink 3, the primary outcome was the change in SBP over 12 months of follow-up as measured by BP values that were routinely collected in clinical encounters and extracted from the EHR. In both trials, the difference in the primary outcome between groups was compared. A focus group of patients with hypertension was conducted in preparation for the Hyperlink 3 trial. This group endorsed BP as the primary outcome of importance to them.

### Analysis

The primary analysis for both trials was according to randomized group (intention-to-treat). In Hyperlink 3, we added a secondary per-protocol analysis to examine the outcomes in patients who were adherent to the intended interventions in the two study arms. In the clinic-based care group, this was defined as a visit with a medical assistant for a BP check within 6 weeks of enrollment. In the telehealth group, this was defined as a visit with a pharmacist or nurse practitioner within 6 weeks of enrollment, sending at least one home BP measurement, and attending a follow-up phone visit with the pharmacist or nurse practitioner.

## Results

### PRECIS-2 scores

**Fig. 1:**
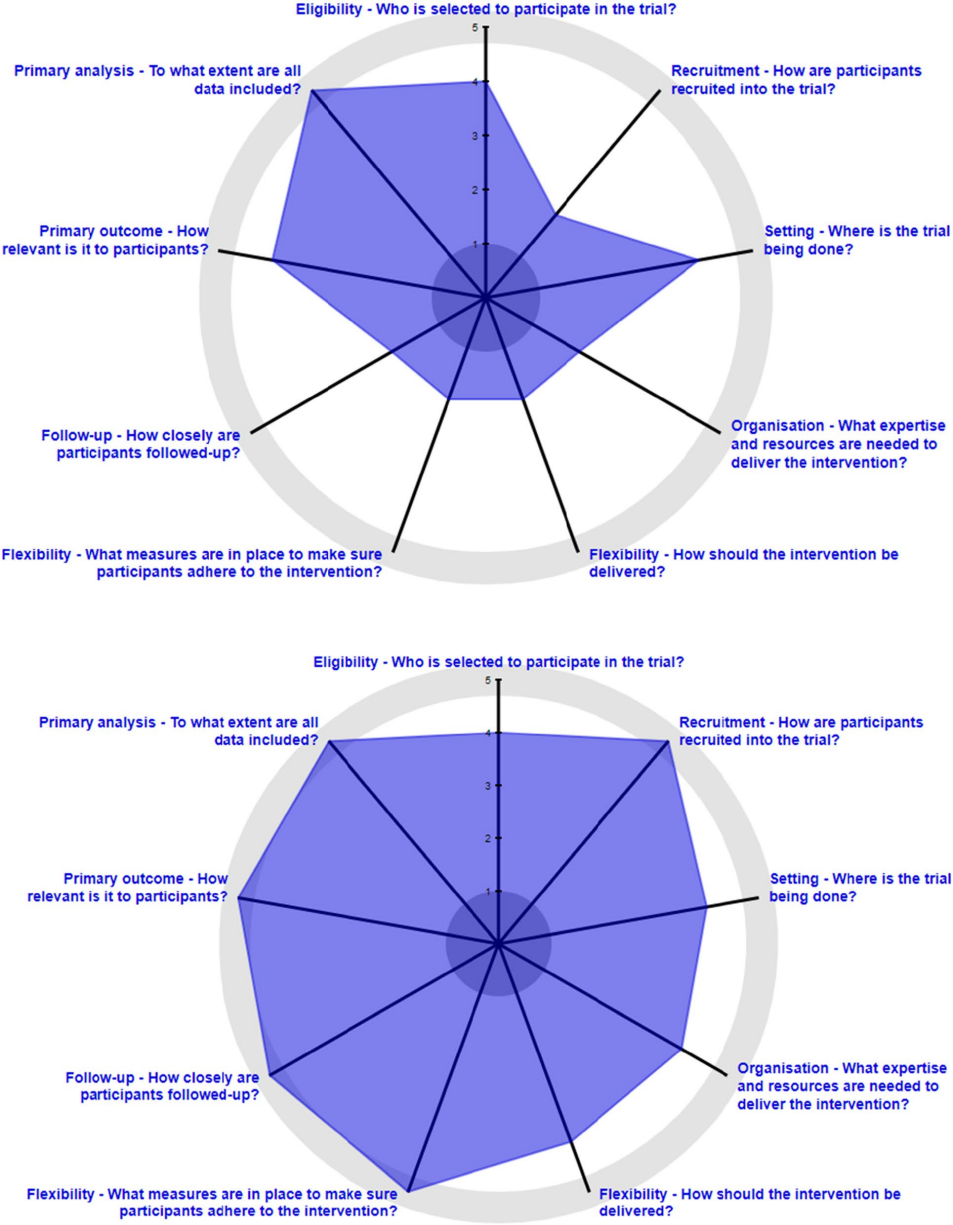
**a** (top) and **b** (bottom) PRECIS-2 wheels for Hyperlink 1 (**a**) and Hyperlink 3 (**b**)

The PRECIS-2 wheels for Hyperlink 1 and 3 are shown in Fig. 1, and the scoring is summarized in Table 2. For the wheels, where only one score is available per domain, if the score differed between the intervention groups, the score for the telemonitoring or telehealth care intervention was used. Both trials were *rather pragmatic* in the setting domain, in that the clinics were representative of routine primary care, but in a single well-resourced integrated health system. Hyperlink 1 recruitment was *rather explanatory* given its reliance on research staff, although some early steps in assessing eligibility were pragmatic in using EHR data to narrow the mailings to patients with elevated BP at recent clinic visits. In contrast, Hyperlink 3 recruitment was *very pragmatic* in its use of automated EHR algorithms; use of existing staff, tools, and processes to enroll patients and arrange follow-up; and partial waiver of informed consent. Both trials were *rather pragmatic* in the eligibility domain in attempting to include typical patients with uncontrolled hypertension and exclude relatively few people except those who required special expertise for hypertension care. Hyperlink 3 had fewer exclusions, but it included only patients with moderately severe uncontrolled hypertension.

**Table 2:** PRECIS-2 domain scores for Hyperlink 1 and Hyperlink 3

PRECIS-2 Domain	Hyperlink 1 score		Hyperlink 3 score	
	Narrative	Numeric	Narrative	Numeric
Setting	Rather pragmatic	4	Rather pragmatic	4
Recruitment	Rather explanatory	2	Very pragmatic	5
Eligibility	Rather pragmatic	4	Rather pragmatic	4
Organization (control)	Very pragmatic	5	Very pragmatic	5
Organization (intervention)	Rather explanatory	2	Rather pragmatic	4
Flexibility of delivery (control)	Very pragmatic	5	Very pragmatic	5
Flexibility of delivery (intervention)	Rather explanatory	2	Rather pragmatic	4
Flexibility of adherence (follow-up)	Very explanatory	1	Very pragmatic	5
Flexibility of adherence (care)	Rather explanatory	2	Rather pragmatic	4
Follow-up	Very explanatory	1	Very pragmatic	5
Primary outcome	Rather pragmatic	4	Very pragmatic	5
Primary analysis	Very pragmatic	5	Very pragmatic	5

In the organization domain, the UC and clinic-based care comparators were very pragmatic. The Hyperlink 1 telemonitoring intervention was paid for by the study, making it *rather explanatory*, while the Hyperlink 3 telehealth care intervention costs were absorbed by the health system, making it *rather pragmatic*. In the flexibility of delivery domain, the UC and clinic-based care comparators were *very pragmatic*. The Hyperlink 1 telemonitoring intervention was more prescriptive and thus *rather explanatory*, while the Hyperlink 3 telehealth care intervention was more tailored to the individual patient and thus *rather pragmatic*. In the flexibility of adherence to intervention domain, Hyperlink 1 UC and Hyperlink 3 clinic-based care and telehealth care were *very pragmatic* for adherence to initial follow-up and subsequent care recommendations. The Hyperlink 1 telemonitoring intervention was *very explanatory* for adherence to initial follow-up, and the pharmacist care was *rather explanatory* in that it was more prescriptive than their typical hypertension care. In contrast, in Hyperlink 3 telehealth care, adherence to initial follow-up was *very pragmatic*, while the pharmacist care was *rather pragmatic* in imposing fewer constraints on their typical hypertension care.

The follow-up domain was *very explanatory* for Hyperlink 1 and *very pragmatic* for Hyperlink 3. The primary outcome domain was *very pragmatic* for Hyperlink 3 based on the patient focus group results and the use of BP measured in routine care as the primary outcome. The primary outcome domain was *rather pragmatic* in Hyperlink 1 because BP measured in a research clinic is less relevant to patients. The primary intention-to-treat analysis was *very pragmatic* for both trials.

### Effect of recruitment methods on enrollment from the potentially eligible populations

**Fig. 2:**
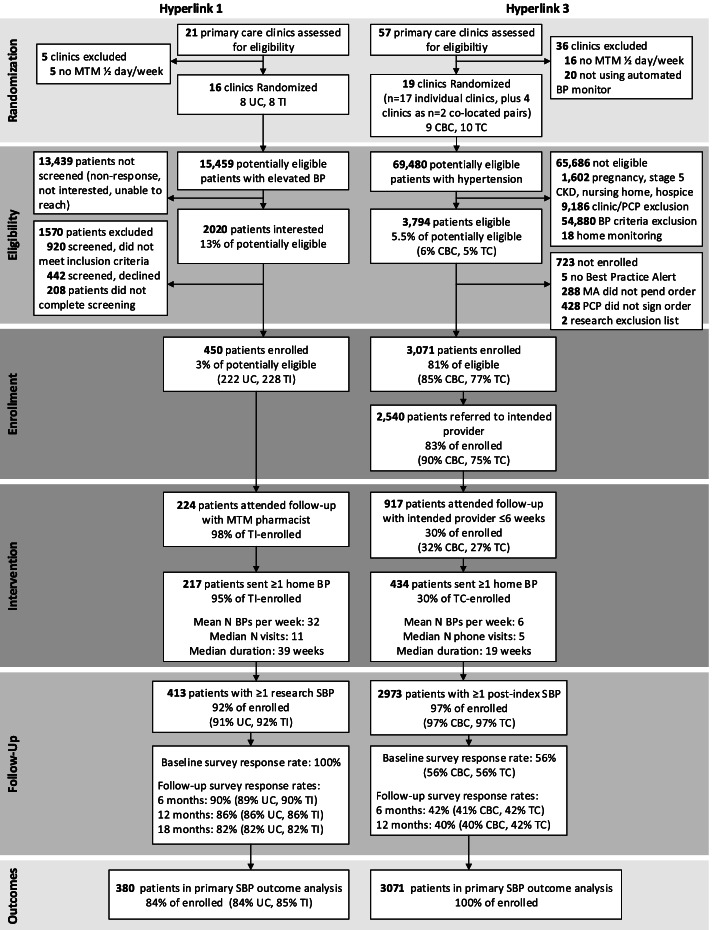
Flow diagram of randomization, eligibility, enrollment, intervention, and follow-up in Hyperlink 1 and Hyperlink 3. Abbreviations: Medication therapy management (MTM), blood pressure (BP), usual care (UC), telemonitoring intervention (TI), clinic-based care (CBC), telehealth care (TC), primary care professional (PCP), medical assistant (MA), chronic kidney disease (CKD), systolic blood pressure (SBP)

In Hyperlink 1, among 15,459 adult patients who were potentially eligible based on age and BP recorded in the EHR and selected for contact by mail, 2020 (13.1%) expressed initial interest in participating and were contacted to be further screened in the research clinic (Fig. 2) [16]. Of these, 920 were excluded (mostly for non-elevated BP), 442 declined, 208 did not complete screening, and 450 were enrolled (2.9% of potentially eligible.)

In Hyperlink 3, among 222,133 patients aged 18–85 seen in 21 primary care clinics (17 individual clinics, plus 4 clinics as 2 co-located pairs) over 18 months, 69,480 patients were potentially eligible based on having two or more encounters with a hypertension diagnosis code within the last 24 months (Fig. 2). Of these patients, 3794 (5.5%) met the additional eligibility criteria, with 1602 (2.3%) having one or more exclusion criteria, 9186 (13.2%) not having been seen by their PCP in the previous 12 months or not being at their assigned PCP’s clinic, and 54,880 (79.0%) not meeting the BP criteria (>150/95 mm Hg) at the current and previous encounter. Of the 3794 eligible patients, 3071 (81%) had a signed hypertension follow-up order and were enrolled. The proportion of eligible patients enrolled was somewhat higher in the clinic-based care group (85%) than in the telehealth group (77%), and for those with signed follow-up orders, PCPs were less likely to change the default follow-up from the intended provider in the clinic-based care group (90% retained medical assistant BP check) than in the telehealth group (75% retained pharmacist).

### Effect of recruitment methods on characteristics of enrolled and non-enrolled patients

Limited data are available on patients who were sent Hyperlink 1 recruitment mailings (Table 3). The enrolled patients were older than the mailing sample (age 61.1 vs. 58.4), less likely to be Asian (1.6% vs. 3.0%) or Black (11.8% vs. 15.2%), and more likely to be male (55.3% vs. 47.0%) and White (81.8% vs.73.0%). Mean BP was similar (148/85 mm Hg).

**Table 3:** Characteristics of patients eligible and enrolled in Hyperlink 1 and 3

	Hyperlink 1 EHR BP eligible	Hyperlink 1 eligible and enrolled	Hyperlink 3 eligible and not enrolled	Hyperlink 3 eligible and enrolled
Total N	15,459	450	723	3071
Age, mean (SD)	58.4 (16.0)	61.1 (12.0)	62.7 (13.6)	60.2 (14.4)
Male, *n* ( %)	7266 (47.0)	249 (55.3)	372 (51.4)	1432 (46.6)
Race				
Asian, *n* ( %)	467 (3.0)	7 (1.6)	40 (5.5)	213 (6.9)
Black, *n* (%)	2350 (15.2)	53 (11.8)	101 (14.0)	594 (19.3)
White, *n* (%)	11,282 (73.0)	368 (81.8)	553 (76.5)	2132 (69.4)
Other/unknown, *n* (%)	1360 (8.8)	22 (4.9)	29 (4.0)	132 (4.3)
Hispanic ethnicity, *n* (%)	303 (2.0)	10 (2.2)	15 (2.1)	60 (2.0)
>4-year college degree, *n* (%)	N/A	209 (47.9)	N/A	526 (31.2)^a^
Employed, *n* (%)	N/A	229 (52.7)	N/A	721 (42.6)^a^
Annual income >$50,000, *n* (%)	N/A	254 (66.5)	N/A	731 (49.4)^a^
SBP, Mean (SD)	147.5 (13.6)	147.9 (13.0)	155.9 (17.1)	158.0 (15.3)
DBP, Mean (SD)	84.8 (12.0)	84.7 (11.6)	88.9 (13.8)	91.7 (14.0)
Antihypertensive medications				
0, *n* (%)	N/A	118 (26.2)	118 (16.3)	465 (15.1)
1, *n* (%)	N/A	116 (25.8)	230 (31.8)	945 (30.8)
2, *n* (%)	N/A	115 (25.6)	197 (27.3)	901 (29.3)
3+, *n* (%)	N/A	101 (22.4)	178 (24.6)	769 (24.8)
BMI >30 kg/m^2^, *n* (%)	N/A	241 (54.3)	367 (51.5)	1730 (57.1)
Diabetes, *n* (%)	N/A	86 (19.1)	197 (27.2)	773 (25.2)
Cardiovascular disease, *n* (%)	N/A	43 (9.6)	139 (19.2)	512 (16.7)

*Abbreviations*: *BP* blood pressure, *EHR* electronic health record, *SBP* systolic blood pressure, *DBP* diastolic blood pressure, *BMI* body mass index

^a^ Survey responses were available for 1688 respondents for education, 1693 for employment status, and 1481 for income

In contrast, in Hyperlink 3, compared with the non-enrolled study-eligible patients, enrolled patients were a few years younger (mean age 60.2 vs. 62.7); more likely to be Asian (6.9% vs. 5.5%) or Black (19.3% vs. 14.0%); and less likely to be male (46.6% vs. 51.4%) and White (69.4% vs. 76.5%). Compared with non-enrolled patients, in the enrolled patients, BP was higher (158/92 mm Hg vs. 156/89 mm Hg); and they were more likely to have obesity (57.1% vs. 51.5%), but less likely to have diabetes (25.2% vs. 27.2%) or cardiovascular disease (16.7% vs. 19.2%).

### Effect of study design on baseline characteristics of enrolled patients

Compared to Hyperlink 1, enrolled patients in Hyperlink 3 were about 1 year younger (60.2 vs. 61.1 years), had a lower proportion of male patients (46.6% vs. 55.3%) and White patients (69.4% vs. 81.8%), and had a higher proportion of Asian patients (6.9% vs. 1.6%) and Black patients, (19.3% vs. 11.8%, Table 3). In Hyperlink 3, socioeconomic indicators were lower than in Hyperlink 1; fewer had earned a college degree (31.2% vs. 47.9%), were employed (42.6% vs. 52.7%), or had an annual household income of $50,000 or more (49.4% vs. 66.5%). Mean BP was higher by design (158/92 vs. 148/85 mm Hg) and somewhat more patients were taking 2 or more antihypertensive medications (54.1% vs. 48.0%). A higher proportion of Hyperlink 3 patients had comorbid conditions.

### Follow-up and adherence of enrolled patients

In Hyperlink 1, we did not measure the timing of follow-up primary care clinic visits in the UC group. Among the 228 telemonitoring intervention group patients in Hyperlink 1, 224 (98%) attended the in-person pharmacist visit and 217 (95%) transmitted at least one BP measurement with the telemonitor (Fig. 2) [24, 25]. They transmitted a mean of about 32 BP measurements per week, and the proportion of weeks with at least 6 readings taken was high (mean 73%, median 81%). Patients attend a median of 11 visits during 39 weeks of follow-up. Adherence to protocol phone visits with the pharmacist in the first 6 months was 88%, and 92% of patients had at least one follow-up BP measured in the research clinic. The proportions of patients attending follow-up research clinic visits and who completed surveys were 90% at 6 months, 86% at 12 months, and 82% at 18 months.

In Hyperlink 3, only 32% of clinic-based care patients had a visit with a medical assistant for a BP check and only 27% of telehealth care enrolled patients had a visit with a pharmacist within 6 weeks of enrollment (Fig. 2). There were 434 (30%) telehealth care patients who eventually saw an MTM pharmacist, agreed to home BP monitoring, and sent in at least 1 home BP measurement, which was more than twice as large as the number of patients who participated in telemonitoring in Hyperlink 1. These patients transmitted a mean of about 6 BP measurements (median 5) per week and attended a median of 5 phone visits with the pharmacist during a median of 19 weeks of follow-up. The proportion of enrolled patients who had >1 post-enrollment BP recorded in the EHR during 24 months of follow-up was 97% in both groups. The proportion who completed the baseline survey was 56%, the 6-month survey was 42%, and the 12-month survey was 39%.

## Discussion

Pragmatic trial designs are widely viewed as a logical next step to demonstrate the effectiveness in real-world settings of clinical interventions that have been shown to be efficacious in explanatory trials. Hyperlink 3 was intentionally designed to follow this progression by incorporating more pragmatic design choices, particularly in the domains of recruitment, resources needed to deliver the telehealth intervention, flexibility of delivery of the telehealth intervention, flexibility of adherence to the intervention, and data collection. The PRECIS-2 tool is increasingly used to compare how explanatory or pragmatic a trial’s design is across nine domains [26, 27]. According to the PRECIS-2 scores, Hyperlink 3 was in fact more pragmatic than Hyperlink 1 in many domains, with the intention to compare the interventions under real-world clinical conditions and avoid the selection effects introduced by using traditional research methods to recruit and consent patients. We aimed to enroll a high proportion of the eligible target population using an EHR algorithm and for the enrolled patients to be highly representative of the target population. In this respect, the trial was successful, enrolling 81% of the eligible patients, whereas the proportion was much lower in Hyperlink 1 (2.9% of potentially eligible patients identified based on BP from the EHR), although the exact denominator of the eligible population cannot be known with certainty.

Hyperlink 3 patients were more representative of the eligible patients than those volunteering for the Hyperlink 1 trial, and the direction of differences tended to favor enrollment of patients who are typically less likely to be included in clinical trials. Enrollees were about 3 years younger, were less likely to be male, were less likely to be White, had higher BP, and had a different pattern of comorbidities than the non-enrolled population. In this unblinded study, these differences are likely to have arisen due to conscious choices on the part of patients and their PCPs for specific types of follow-up care. This contrasts with results often seen in explanatory trials and the direction of selection in the Hyperlink 1 study, which tended to favor enrollment of older patients, men, and White patients. We suspect that Hyperlink 1 enrollment was more influenced by patient preferences for participation in a research study than for specific types of care.

One major departure from Hyperlink 1 was the much higher BP required for Hyperlink 3 eligibility (>150/95 mm Hg at 2 consecutive visits), a consequence of the semi-automated method of enrollment and the very large number of patients who would have been eligible at the lower BP threshold in Hyperlink 1 (>140/90 mm Hg at 2 consecutive visits). This type of choice is one often faced by both pragmatic trialists and health system leaders faced with limited resources and large populations in need of care. The higher BP for eligibility in Hyperlink 3 may have had some effect on the characteristics of the enrolled population, but it seems likely that the greater inclusiveness of the recruitment method and the waiver of the informed consent process were the primary drivers of the more vulnerable population enrolled in Hyperlink 3 (more Asian or Black, lower educational attainment, employment, and annual income, and more likely to have comorbidities.)

Another aspect of the Hyperlink 3 design that deserves mention is the choice to define enrollment as the point where a hypertension referral order was signed by the PCP. This choice was in keeping with the tenet that pragmatic trials should reflect the realities of actual patients, clinicians, and care settings, our goal to have the enrolled population closely reflect the eligible population, and the need to enroll comparable patients from both randomized groups. Even so, there was a difference in the proportion enrolled in the clinic-based care group (85%) and the telehealth group (77%), likely because physicians or patients preferred accepting the default follow-up with the more familiar medical assistant. Although the study team had expected moderately lower adherence to the telehealth intervention than the 98% observed in the highly motivated and consented patients in Hyperlink 1, we were surprised by the very low adherence to the hypertension referral follow-up visits in both groups in Hyperlink 3. Hyperlink 3 patients also sent in fewer home BP measurements than Hyperlink 1 patients (mean 32/week vs. 6/week).

Although the low-touch recruitment methods in Hyperlink 3 succeeded in enrolling a larger and more representative sample of patients with uncontrolled hypertension without using traditional research methods and staff, these patients were much less likely to adhere to the follow-up visit, and thus less likely to be exposed to the telehealth intervention. Although the PCPs signed the referral order for follow-up, we do not know if they discussed it with the patients or encouraged it. Only routine clinic resources were used for scheduling follow-up. There was no written consent to participate in the study, further reducing patient awareness of the follow-up care recommended for them. The low participation rate we observed is likely to dilute any positive effect of the intervention as assessed by the gold standard of intention-to-treat analysis. In retrospect, we should have anticipated this challenge based on other pragmatic trials that were launched at around the same time [28–30]. However, it also means that Hyperlink 3 results provide a much more realistic picture of what others are likely to find if they recruit unselected patients with poorly controlled BP and direct them to a single preferred option for the type of follow-up care. A design option that we did not consider for Hyperlink 3, but perhaps should receive more consideration for future pragmatic trials, would allow patients more flexibility and freedom of choice within randomized treatment groups (e.g., telehealth care overseen by the PCP without involvement of a pharmacist, home BP monitoring with other methods of transmitting BP, or stepped care approaches). It is also worth questioning whether minimal attention to adherence, and thus a higher PRECIS-2 score in that domain, is always desirable for pragmatic trials. Rather, it may make sense to take a balanced approach to key mediators of intervention effect, which for this intervention includes sufficient levels of self-monitoring of BP and a focus on medication intensification [31].

These observations also suggest that in pragmatic trials, the primary intention-to-treat analysis demonstrates the real-world effect of offering the intervention widely when intervention adherence and fidelity are promoted as they would be outside a trial. The analysis may be supplemented by a secondary per-protocol analysis that compares results in patients who adhered to the intervention in both groups, using propensity weights to take into account the post-randomization differences between groups [32]. We have planned to do this type of analysis, which differs from simple unadjusted comparisons of patients who participated in or completed an intervention. Moreover, the observed rate of adherence to the intervention in those who were eligible is a key piece of information for those who may be considering use of a given intervention strategy in other settings or patient populations. If the adherence or uptake rate is low in a broad class of patients who could potentially benefit, but if costs are low for offering it to patients who do not avail themselves of the intervention, it may still make sense as an option for health systems to improve hypertension care. Regardless of whether a per-protocol analysis shows greater intervention effects than the intention-to-treat analysis, further studies will be needed to learn how to increase participation in a voluntary telehealth intervention and whether payors and care systems are willing to subsidize these extra costs.

## Conclusions

The pragmatic design of Hyperlink 3 was successful in enrolling a high proportion of eligible patients and those traditionally under-represented in clinical trials (women, minorities, and patients with less education and lower income), and demonstrating that this could be done without research staff using an EHR algorithm. However, there were trade-offs with much lower adherence than in Hyperlink 1. The lower adherence is likely to greatly reduce any differential intervention effect on the primary outcome, systolic BP, and other secondary outcomes in the more pragmatic Hyperlink 3 study design but likely more accurately demonstrates real-world outcomes associated with an intervention of this type. It will require careful interpretation and supplementation of the intention-to-treat analysis with a planned per-protocol analysis. Nevertheless, for health care systems interested in implementing similar interventions in real-life settings, Hyperlink 3 provides them with a much better estimate of the uptake and an opportunity to devise additional ways to encourage participation. Moreover, these observations support the hypothesis that to improve care of common chronic diseases a variety of intervention or care improvement models may need to be employed so that patients have more than one pathway to improved care.

## Data Availability

The datasets used and/or analyzed during the current study are available from the corresponding author on reasonable request.

## Abbreviations

BP: Blood pressure
BMI: Body mass index
CBC: Clinic-based care
CKD: Chronic kidney disease
DBP: Diastolic blood pressure
EHR: Electronic health record
IRB: Institutional review board
MTM: Medication therapy management
PCP: Primary care professional
PRECIS-2: PRagmatic Explanatory Continuum Indicator Summary-2
SBP: Systolic blood pressure
TC: Telehealth care
TI: Telemonitoring intervention
UC: Usual care
